# C-Peptide Is Independently Associated with an Increased Risk of Coronary Artery Disease in T2DM Subjects: A Cross-Sectional Study

**DOI:** 10.1371/journal.pone.0127112

**Published:** 2015-06-22

**Authors:** Lingshu Wang, Peng Lin, Aixia Ma, Huizhen Zheng, Kexin Wang, Wenjuan Li, Chuan Wang, Ruxing Zhao, Kai Liang, Fuqiang Liu, Xinguo Hou, Jun Song, Yiran Lu, Ping Zhu, Yu Sun, Li Chen

**Affiliations:** 1 Department of Endocrinology, Qilu Hospital of Shandong University, Jinan, Shandong 250012, China; 2 Department of General Surgery, Qilu Hospital of Shandong University, Jinan, Shandong 250012, China; 3 Department of Ophthalmology, College of medicine, University of Florida, Gainesville, FL 32610–0284, United States of America; 4 Institute of Endocrinology and Metabolism, Shandong University, Jinan, Shandong 250012, China; University of Catanzaro Magna Graecia, ITALY

## Abstract

**Objective:**

C-peptide has been reported to be a marker of subclinical atherosclerosis in type 2 diabetes mellitus (T2DM) patients, whereas its role in coronary artery disease (CAD) has not been clarified, especially in diabetics with differing body mass indices (BMIs).

**Design and Methods:**

This cross-sectional study included 501 patients with T2DM. First, all subjects were divided into the following two groups: CAD and non-CAD. Then, binary logistic regression was used to determine the risk factors for CAD for all patients. To clarify the role of obesity, we re-divided all subjects into two additional groups (obese and non-obese) based on BMI. Finally, binary logistic regression was used to determine the risk factors for CAD for each weight group.

**Results:**

The patients with CAD showed a higher BMI and fasting C-peptide level in addition to an increased prevalence of traditional risk factors for CAD, such as hypertension, insulin resistance, higher cholesterol, cysteine-C (Cys-C) and lower estimated glomerular filtration rate (eGFR). Logistic regression analysis showed that fasting C-peptide (OR=1.513, p=0.005), insulin treatment (OR=1.832, p=0.027) hypertension (OR=1.987, p=0.016) and hyperlipidemia (OR=4.159, p<0.001) significantly increased the risk of clinical CAD in the T2DM patients independent of age, gender, diabetes duration, smoking and alcohol statuses, fasting insulin and glucose, hypoglycemic episodes, UA and eGFR. Additionally, in both of the obese (OR=1.488, p=0.049) and non-obese (OR=1.686, p=0.037) DM groups, C-peptide was associated with an increased risk of CAD after multiple adjustments.

**Conclusions:**

C-peptide is associated with an increased CAD risk in T2DM patients, no matter whether they are obese or not.

## Introduction

Recently, diabetes mellitus has emerged as a global and regional health problem with a rapidly increasing prevalence [1,2]. T2DM is an independent risk factor of CAD [3], and cardiovascular disease has become the leading cause of mortality among diabetic patients [4]. Patients with diabetes display a higher CAD prevalence, worse cardiac outcomes and more severe coronary atherosclerosis than those with normal glycemic levels [5]. In fact, the co-occurrence of CAD and T2DM may be attributed to several common conditions, such as chronic inflammation [6], insulin resistance [7,8], oxidative stress [9] and metabolic disorders [10], and diabetes further aggravates these conditions and contributes to atherosclerotic plaque formation. However, the involvement of other factors in addition to the above-mentioned traditional risk factors for CAD remains to be elucidated.

Initially, C-peptide was considered to be an inactive peptide and was used as a marker of insulin secretion. It was not until the past few decades that researchers have discovered that this peptide appears to have a protective effect on T1DM-associated microvascular complications [11]. However, in T2DM patients, studies have indicated that the C-peptide might exhibit some proinflammatory properties in the process of atherosclerotic plaque formation. A study performed by Walcher and colleagues has indicated that C-peptide accumulates in the subendothelial space and intima [12] and may induce smooth muscle cell proliferation [13]. In addition, basal C-peptide levels have also been significantly correlated with the intima-media thickness of the carotid artery in T2DM patients, suggesting that it could be a surrogate marker of subclinical atherosclerosis [14]. These data suggest that the C-peptide might participate in the inflammatory response and contribute to the development of CAD in T2DM patients. However, its role in clinical atherosclerosis in T2DM patients has not yet been clarified. Furthermore, considering the obese diabetic patients typically exhibit a higher C-peptide level, and they also have a higher risk of CAD and a worse prognosis than those who are of normal weight [15–17], whether C-peptide may act differently in the different weight groups with T2DM warrants further examination.

Here, to address the issues discussed above, subjects were evaluated for an association between C-peptide and the risk of CAD and then divided into 2 groups based on BMI. The role of C-peptide in the occurrence of CAD in obese diabetic (Ob-DM) and non-obese diabetic (Nob-DM) patients was explored.

## Materials and Methods

### Subjects

This cross-sectional study randomly recruited 539 subjects diagnosed with T2DM at Qilu Hospital of Shandong University from April 2012 to July 2013. The following exclusion criteria were applied: patients with 1) missing data for calculating the BMI; 2) T1DM, secondary diabetes or specific types of diabetes or diabetic ketoacidosis, lactic acidosis or a hyperglycemic hyperosmolar state; 3) DM foot or inflammatory or infectious diseases; 4) familial hypercholesterolemia or valvular disease, myocardial disease or heart failure; and 5) severely impaired liver or renal function. Finally, a total of 501 subjects (235 women) were eligible for the study. Written informed consent was obtained from all subjects, and the study was approved by the ethics committee of the Qilu Hospital of Shandong University.

### Clinical Evaluation

The computerized patient record system of Qilu Hospital was used to collect data regarding the demographic characteristics, lifestyles and previous medical histories of the subjects. Anti-diabetic medications were included in the following categories: insulin, insulin secretagogues and others(thiazolidinediketones, metformin and alpha glucosidase inhibitor). Body mass index (BMI) was determined by dividing the weight by the height squared (kg/m2). Blood pressure (BP) was measured 3 consecutive times (OMRON Model HEM-752 FUZZY, Omron Company, Dalian, China) using the left arm after the subject had remained seated for at least 5 min, and the average reading was used for the analysis. Blood samples were collected after a 10-hour fast and before the ingestion of breakfast and medication. Patients taking neutral protamine Hagedorn (NPH) or insulin glargine were switched to sulfonylurea (1.25 or 2.5 mg glibenclamide) on the night before sampling. Fasting glucose, total cholesterol (TC), triglycerides (TGs), low-density lipoprotein cholesterol (LDL-C), high-density lipoprotein cholesterol (HDL-C), nonesterified fatty acids (NEFAs), blood urea nitrogen (BUN), creatinine (Cr), uric acid (UA) and cysteine-C (Cys-C) levels were measured by an automatic analyzer (TOSHIBA TBA-40F, Toshiba, Japan). HbA1C was measured by high-performance liquid chromatography (BIO–RAD VARIANT II, Bio-Rad, USA). Blood insulin and C-peptide levels were detected by a chemiluminesence immunoassay analyzer (Bayer ADVIA Centaur, Bayer, Germany). Albuminuria was detected by an immunoturbidimetric assay analyzer (Siemens BN ProSpec System, Siemens, Germany). Insulin resistance was estimated by the classic homeostasis model assessment index of IR (HOMA-IR) developed by Mathew [18] using the following formula: HOMA1-IR = fasting plasma insulin (μU/mL) x fasting plasma glucose (mmol/L)/22.5. The eGFR was calculated using creatinine level according to the Chronic Kidney Disease Epidemiology Collaboration (CKD-EPI) equation[19], and mild-moderate kidney dysfunction was defined as a eGFR≥30 mL/min/1.73 m^2^ and<60 mL/min/1.73 m^2^ according to the Kidney Disease Outcomes Quality Initiative (K/DOQI) clinical practice guidelines[20].

### Definitions

A BMI≥25 kg/m^2^ was defined as obesity, according to the criteria of the World Health Organization (WHO) Asia-Pacific obesity classification [21].

Diabetic patients who has been previously diagnosed were identified after a review of their medical records based on the following 1999 WHO criteria: FPG≥126 mg/dL (7.0 mmol/L) and/or 2hPG≥200 mg/dL (11.1 mmol/L) [22].

CAD was diagnosed in the Department of Cardiology at Qilu Hospital, according to the following criteria: 1) angina; 2) ischemic ECG; or 3) hard CAD. Hard CAD was defined as a history of myocardial infarction (MI) confirmed by ECG Q waves or medical records, coronary revascularization, or coronary artery occlusion of ≥50% as shown by angiography [23].

Hypertension was defined as blood pressure ≥140/90 mmHg or current antihypertensive treatment. Dyslipidemia was defined as low HDL and/or high LDL. Low HDL was defined as an HDL-C level of <0.9 mmol/L (men) or <1.0 mmol/L (women). High LDL was defined by the daily intake of statins or an LDL of >4 mmol/L [9].

Hypoglycemia episode was defined as a recorded plasma glucose value of 70 mg/dl (3.9 mmol/L) or less[24].

### Statistical Analysis

The normally distributed continuous variables are expressed as the mean±standard deviation (SD), and the variables with non-normal distribution are presented as the median (25th percentile-75th percentile). The categorical variables are presented as numbers (%).

A P<0.05 was considered statistically significant. Differences between two groups were evaluated using Student’s t test and the χ 2 test, and for three groups or more, one-way ANOVA was applied. Non–normally distributed variables (e.g., Cr, BUN and fasting insulin) were log10 transformed, and the Mann-Whitney U test was used to compare continuous variables that could not be log normalized. Binary logistic regression was used to determine the risk factors for CAD in the T2DM patients. All of the above statistical analyses were performed with SPSS 16.0 software.

## Results

### Characteristics overall and of the Nob-DM and Ob-DM subjects

The demographics and lab results of all participants are shown in Table 1. Among all subjects, 58.08% (291/501) were diagnosed with CAD. The patients with CAD showed a longer history of T2DM and higher BMI and increases in insulin usage, the incidence of hypertension, and the frequency of statin treatment. Traditional risk factors for CAD, such as insulin resistance, cholesterol and Cys-C, also showed higher levels, together with an elevation in the fasting C-peptide level and a decline in the eGFR. Next, we compared the clinical characteristics of the Nob-DM (n = 210) and Ob-DM (n = 291) groups separately for the CAD and non-CAD patients, and the results are listed in Table 2. In the non-obese subjects, the DM duration, incidence of hypertension, incidence of hypoglycemia episodes and insulin usage were higher in the CAD group, whereas these differences were not significant between the groups in the obese subjects. The C-peptide, cholesterol and Cys-C levels were generally higher in both the obese and non-obese CAD patients.

**Table 1:** Bioclinical characteristics of non-CAD diabetic and CAD diabetic subjects.

	non-CAD (n = 210)	CAD (n = 291)	*P*
**Women [n (%)]**	98(46.7%)	137(47.1%)	0.927
**Age (years)**	65±8	66±9	0.065
**DM history (years)**	10.07±7.52	12.28±7.67	**0.001**
**BMI (kg/m** ^**2**^ **)**	25.19±3.36	26.12±3.71	**0.004**
**Hypertension [n (%)]**	118(56.2%)	214(73.5%)	**<0.001**
**SBP (mmHg)**	143±20	142±19	0.827
**DBP (mmHg)**	78±12	78±13	0.502
**Glucose (mmol/L)**	8.04±2.67	8.33±2.90	0.264
**Insulin (mU/mL)**	8.90(5.68–14.36)	10.20(5.60–16.00)	**0.026**
**C-peptide (ng/mL)**	1.34(0.88–1.94)	1.73(1.13–2.48)	**<0.001**
**HOMA-IR**	2.87(1.85–4.89)	3.35(1.99–5.89)	**0.011**
**HbA1c (%)**	8.28±1.90	8.41±1.87	0.453
**Insulin secretagogues treatment [n (%)]**	107(51.0%)	151(52.1%)	0.805
**Other anti-diabetic medications [n (%)]**	140(66.7%)	175(60.3%)	0.148
**Insulin treatment [n (%)]**	85(40.5%)	159(54.6%)	**0.001**
**Hypoglycemic episodes[n (%)]**	27(12.9%)	45(15.5%)	0.333
**Total cholesterol (mmol/L)**	5.04±1.09	4.69±1.19	**0.001**
**TGs (mmol/L)**	1.59±1.02	1.71±1.18	0.266
**HDL-c (mmol/L)**	1.31±0.35	1.18±0.30	**<0.001**
**LDL-c (mmol/L)**	3.01±0.86	2.81±0.91	**0.012**
**Statin treatment [n (%)]**	31(14.8%)	166(57.0%)	**<0.001**
**NEFAs (μmol/dL)**	53.14±22.33	59.81±23.59	**0.005**
**UA (μmol/L)**	284.03±86.33	299.80±96.46	0.070
**Cys-C (mg/L)**	0.95(0.82–1.12)	1.03(0.89–1.23)	**<0.001**
**eGFR (mL/min/1.73 m** ^**2**^ **)**	91.42±20.75	84.63±22.80	**0.001**
**Mild-moderate kidney dysfunction[n (%)]**	15(7.3%)	30(10.4%)	0.232
**Albuminuria (mg/L)**	11(10.5–62)	14.3(11–54.5)	**0.025**
**Smoking status[n (%)]**	52(24.8%)	56(19.2%)	0.138
**Alcohol consumption[n (%)]**	52(24.8%)	62(21.3%)	0.363

Data are shown as the mean ± SD, median (interquartile range) or number (%).

SBP, systolic blood pressure; DBP, diastolic blood pressure; TC, total cholesterol; TGs, triglycerides; LDL-C, low-density lipoprotein cholesterol; HDL-C, high-density lipoprotein cholesterol; NEFAs, nonesterified fatty acids; BUN, blood urea nitrogen; Cr, creatinine; UA, uric acid; Cys-C, cysteine-C and eGFR, estimated glomerular filtration rate.

**Table 2:** Bioclinical characteristics of non-obese diabetic (Nob-DM) and obese diabetic (Ob-DM) subjects.

	Nob-DM (n = 210)		Ob-DM (n = 291)	
	non-CAD	CAD	non-CAD	CAD
**Women [n (%)]**	47(43.5%)	52(51.0%)	51(50.0%)	85(45.0%)
**Age (years)**	66±9	66±8	64±8	**66±9** *
**DM history (years)**	9.24±7.56	**10.00(7.00–17.00)** *	10.94±7.41	11.00(6.00–20.00)
**BMI (kg/m** ^**2**^ **)**	22.59±1.90	22.52±2.92	27.95±2.15	28.06±2.41
**Hypertension [n (%)]**	50(46.3%)	**68(66.7%)** *	68(66.7%)	146(77.2%)
**SBP (mmHg)**	140(129–157)	**137±19** *	141(130–154)	145±18
**DBP (mmHg)**	77±12	**74±12** *	80±12	80±13
**Glucose (mmol/L)**	7.70±2.64	8.00±2.61	8.41±2.66	8.50±3.03
**Insulin (mU/mL)**	9.63±7.06	11.67±10.40	12.10±7.94	13.87±11.44
**C-peptide (ng/mL)**	1.33±0.79	**1.78±1.05** **	1.70±0.92	**1.95±1.01** *
**HOMA-IR**	2.45(1.67–3.59)	4.02±4.42	3.40(2.19–6.01)	5.05±4.24
**HbA1c (%)**	7.90(6.65–9.70)	8.26±1.77	7.80(6.90–9.55)	8.49±1.93
**Insulin secretagogues treatment [n (%)]**	58(53.7%)	52(51.0%)	49(48.0%)	99(52.7%)
**Other anti-diabetic medications [n (%)]**	71(65.7%)	60(58.8%)	69(67.6%)	115(61.2%)
**Insulin treatment [n (%)]**	39(36.1%)	**60(58.8%)** *	46(45.1%)	99(52.9%)
**Hypoglycemic episodes[n (%)]**	13(12.0%)	**25(24.5%)** *	14(13.7%)	20(10.6%)
**Total cholesterol (mmol/L)**	5.06±1.14	**4.66±1.08** *	5.03±1.04	**4.70±1.24** *
**TGs (mmol/L)**	1.39±0.90	1.58±1.23	1.81±1.11	1.77±1.15
**HDL-c (mmol/L)**	1.36±0.30	**1.21(0.96–1.40)** *	1.25±0.39	**1.10(0.98–1.30)** *
**LDL-c (mmol/L)**	3.06±0.86	**2.73±0.86** *	2.97±0.87	2.84±0.94
**Statin treatment [n (%)]**	15(13.9%)	**62(60.8%)** *	16(15.7%)	**104(55.0%)** **
**NEFAs (μmol/dL)**	53.92±22.77	60.95±25.17	52.41±22.01	**59.13±22.68** *
**UA (μmol/L)**	264.62±72.32	**288.45±104.81** *	303.83±94.95	305.70±91.58
**Cys-C (mg/L)**	1.00±0.36	**1.14±0.44** *	0.99±0.24	**1.09±0.36** *
**eGFR (mL/min/1.73 m** ^**2**^ **)**	90.44±18.68	85.46±21.64	92.45±22.76	**84.19±23.43** *
**Mild-moderate kidney dysfunction[n (%)]**	8(7.6%)	8(8.1%)	7(6.9%)	22(11.6%)
**Albuminuria (mg/L)**	11.0(10.5–38.0)	11.0(11.0–26.4)	14.0(10.5–87.0)	19.3(11.0–60.4)
**Smoking status[n (%)]**	26(24.1%)	18(17.6%)	26(25.5%)	38(20.1%)
**Alcohol consumption[n (%)]**	25(23.1%)	19(18.6%)	27(26.5%)	43(22.8%)

Data are shown as the mean ± SD, median (interquartile range) or number (%).

*p<0.05 vs. non-CAD subjects.

**p<0.001 vs. non-CAD subjects.

SBP, systolic blood pressure; DBP, diastolic blood pressure; TC, total cholesterol; TGs, triglycerides; LDL-C, low-density lipoprotein cholesterol; HDL-C, high-density lipoprotein cholesterol; NEFAs, nonesterified fatty acids; BUN, blood urea nitrogen; Cr, creatinine; UA, uric acid; Cys-C, cysteine-C and eGFR, estimated glomerular filtration rate.

### Binary logistic regression analysis of related risk factors for CAD

To explore the relationship between C-peptide and CAD in T2DM patients, we first conducted logistic regression analysis using 4 models to assess our total population, as shown in Table 3. With model 1, although no adjustments were made for any other risk factors, C-peptide significantly increased the risk of clinical CAD in the T2DM patients. After adjusting for age, gender and BMI in model 2, fasting C-peptide still showed a strong impact on the incidence of CAD. To explore the role of risk factors associated with T2DM in this context, we adjusted for duration of T2DM, history of insulin treatment, insulin secretagogues or other anti-diabetic medications, hypoglycemia episodes, fasting serum insulin and fasting glucose in model 3. As expected, an increase in the level of fasting C-peptide significantly increased the risk of CAD independent of BMI, insulin resistance, glycemic control, hypoglycemic therapy, incidence of hypoglycemia and disease duration. To further analyze the role of traditional CAD risk factors, such as smoking, drinking, hypertension, dyslipidemia, UA and eGFR, in this association, we performed additional adjustments for these factors in model 4. The increasing level of fasting C-peptide was still significantly associated with an increased risk of CAD in this model, and insulin treatment, hypertension and hyperlipidemia were also shown to lead to a significantly increased risk.

**Table 3:** Binary logistic regression analysis of the associations between different risk factors for CAD.

Characteristics	Model 1		Model 2		Model 3		Model 4	
	OR(95% CI)	*P*	OR(95% CI)	*P*	OR(95% CI)	*P*	OR(95% CI)	*P*
**Fasting C-peptide**	**1.536(1.248–1.891)**	**<0.001**	**1.477(1.194–1.826)**	**<0.001**	**1.586(1.228–2.047)**	**<0.001**	**1.513(1.135–2.017)**	**0.005**
**Age**			1.022(1.000–1.045)	0.053	1.008(0.981–1.036)	0.560	0.994(0.961–1.027)	0.711
**Gender**			0.981(0.671–1.436)	0.922	0.766(0.487–1.206)	0.249	0.832(0.459–1.509)	0.545
**BMI**			**1.058(1.002–1.117)**	**0.041**	1.032(0.969–1.098)	0.326	1.028(0.956–1.106)	0.457
**DM duration**					1.031(0.998–1.066)	0.067	1.010(0.973–1.049)	0.592
**Insulin secretagogues treatment**					1.200(0.730–1.972)	0.473	1.270(0.726–2.223)	0.403
**Other anti-diabetic medications**					0.973(0.587–1.611)	0.914	0.905(0.511–1.603)	0.731
**Insulin treatment**					1.539 (0.953–2.485)	0.078	**1.832(1.070–3.138)**	**0.027**
**Fasting glucose**					1.047(0.963–1.138)	0.280	1.017(0.925–1.119)	0.724
**Fasting plasma insulin**					1.006(0.981–1.032)	0.629	0.999(0.971–1.027)	0.920
**Hypoglycaemic episodes**					1.485(0.793–2.781)	0.216	1.702(0.833–3.477)	0.145
**Smoking status**							1.143(0.568–2.299)	0.708
**Alcohol consumption**							1.095(0.530–2.264)	0.806
**Hypertension**							**1.987(1.135–3.480)**	**0.016**
**Dyslipidemia**							**4.159(2.492–6.940)**	**<0.001**
**UA**							0.999(0.996–1.002)	0.422
**eGFR**							0.995(0.982–1.008)	0.467

Model 1: not adjusted

Model 2: adjusted for age, gender and BMI

Model 3: adjusted for age, gender, BMI, duration of T2DM, history of insulin secretagogues treatment, insulin treatment and other anti-diabetic medications, fasting glucose, fasting plasma insulin and hypoglycaemic episodes

Model 4: adjusted for age, gender, BMI, duration of T2DM, history of insulin secretagogues treatment, insulin treatment and other anti-diabetic medications, fasting glucose, fasting plasma insulin, hypoglycaemic episodes, smoking and drinking statuses, UA, eGFR, hypertension and dyslipidemia

UA, uric acid; eGFR, estimated glomerular filtration rate

Next, to explore whether C-peptide played a role in CAD in the obese and non-obese subjects, we repeated the same binary logistic regression analysis for the Nob-DM and Ob-DM groups, as shown in Table 4. In the non-obese DM subjects, fasting C-peptide significantly increased the risk of CAD after adjusting the above-mentioned risk factors. Moreover, in the obese subjects, the increase in the level of fasting C-peptide also significantly increased the risk of CAD independent of age, gender, BMI and traditional risk factors associated with T2DM and CAD.

**Table 4:** Binary logistic regression analysis of the associations between different risk factors for CAD in obese and normal-weight subjects.

		Nob-DM		Ob-DM	
Model	Independent variable	OR(95% CI)	*P*	OR(95% CI)	*P*
**Model 1**	**C-peptide, ng/mL**	1.734(1.242–2.421)	0.001	1.311(1.002–1.715)	0.049
**Model 2**	**C-peptide, ng/mL**	1.767(1.258–2.483)	0.001	1.303(0.989–1.715)	0.060
**Model 3**	**C-peptide, ng/mL**	1.872(1.240–2.826)	0.003	1.435(1.014–2.031)	0.041
**Model 4**	**C-peptide, ng/mL**	1.686(1.031–2.757)	0.037	1.488(1.002–2.210)	0.049

Model 1: not adjusted

Model 2: adjusted for age, gender and BMI

Model 3: adjusted for age, gender, BMI, duration of T2DM, history of insulin secretagogues treatment, insulin treatment and other anti-diabetic medications, fasting glucose, fasting plasma insulin and hypoglycaemic episodes

Model 4: adjusted for age, gender, BMI, duration of T2DM, history of insulin secretagogues treatment, insulin treatment and other anti-diabetic medications, fasting glucose, fasting plasma insulin, hypoglycaemic episodes, smoking and drinking statuses, UA, eGFR, hypertension and dyslipidemia

UA, uric acid; eGFR, estimated glomerular filtration rate

## Discussion

In this study, we first compared the clinical characteristics of the CAD and non-CAD subjects in our general population followed by a comparison of those in the Nob-DM and Ob-DM groups. Some known risk factors of CAD such as dyslipidemia, arterial hypertension[9], mild-moderate declined eGFR[25], hypoglycaemic episodes[26], hyperinsulinemia[23] and hypoglycemic medications[27] were also included. Similar to what has been previously reported, the patients with CAD showed a longer duration of T2DM, a higher incidence of hypertension, and increases in insulin usage and the frequency of statin treatment in addition to elevated levels of traditional CAD risk factors, such as dyslipidemia, Cys-C and a declined level of eGFR. It is noteworthy that the occurrence of mild-moderate kidney dysfunction was generally low(<10% of each group) in our population, so its influence on CAD might have been undermined. This may explain the lack of statistical significance of the relationship between eGFR and CAD.

A large proportion of diabetic patients are obese, and high BMI itself is an important CAD risk factor[28]. So after evaluating the effects of C-peptide on clinical CAD in the general population, we then sought to find out whether C-peptide may act differently in the different weight groups with T2DM. In the general population, obese as well as the non-obese group, C-peptide was correlated with CAD independent of traditional CAD risk factors and risk factors associated with T2DM, such as age, gender, smoking status, insulin resistance and glycemic control. These results suggest that C-peptide may be associated with or be involved in the process of atherosclerotic plaque formation in the coronary arteries of T2DM patients, no matter whether they are obese or not.

C-peptide has long been believed to lack biological activity. However, recent studies have revealed that it displays various functions in different cell types, although its receptor is unknown. These functions include binding to the pertussis-toxin-sensitive G-protein-coupled receptor on Swiss 3T3 fibroblasts, activation of the p38 protein kinase pathway in mouse lung capillary endothelial cells and activation of Na+/K+-ATPase in renal tubule segments [29]. At physiological concentrations, C-peptide seems to have a positive effect on chronic complications associated with T1DM, such as diabetic neuropathy and endothelial dysfunction, by providing protection against glucose-induced endothelial apoptosis. Similar effects have also been observed in the aortic tissues of streptozotocin diabetic mice following the administration of C-peptide at an approximately 20-fold greater level than the normal exogenous C-peptide level in vivo [11]. However, the effects of this peptide on T2DM patients and cell proliferation are controversial. According to previous studies, C-peptide contributes to plaque formation by accumulating in the subendothelial space and the intima of diabetic subjects [12], attracting both monocytes and CD4+ lymphocytes in a concentration-dependent manner [23] and inducing smooth muscle cell proliferation via Src kinase, phosphatidylinositol 3-kinase and extracellular signal-regulated kinase 1/2 [13]. In the current cross-sectional study, C-peptide was found to be correlated with CAD independent of traditional CAD risk factors, such as age, hypertension, smoking and dyslipidemia, supporting the results of the in vitro experiments as well as correlating with the results of previous population-based studies. C-peptide has previously been reported to be strongly related to the increased incidences of cardiovascular-related and overall deaths in non-diabetic patients[30–33]. In T2DM patients, basal C-peptide has been significantly correlated with the intima-media thickness of the carotid artery, suggesting that it could be a surrogate marker of subclinical atherosclerosis [14]. Our findings increase the current understanding of C-peptide function, and we suggest that this protein may be useful as a marker of clinical atherosclerosis in T2DM patients, especially those who are obese.

There were several limitations of our study. First, the cross-sectional design did not allow for us to explore the temporal relationship between the serum C-peptide level and the development of coronary atherosclerosis; therefore, further longitudinal studies are needed. Second, in our study, insulin resistance was measured by the homeostasis model assessment (HOMA), while the gold standard for insulin resistance is the hyperinsulinemic euglycemic clamp technique. However, considering the complicated procedure and high cost of the latter, we chose HOMA-IR to measure insulin resistance in our subjects. Third, other inflammatory factors (such as CRP, IL-6, E-selectin, ICAM-1, VCAM-1, and PAI-1) that are known to be related to cardiovascular risk were not measured, which may have led to a lack of explanatory power of the role of C-peptide as an atypical inflammatory marker of CAD. Finally, because our subjects were enrolled from a single center, there were unavoidable biases associated with patient selection, the information obtained, and the confounding variables.

In conclusion, we found that C-peptide is correlated with clinical CAD independent of traditional CAD risk factors, such as age, hypertension, smoking status, dyslipidemia, glycemic control and insulin resistance, in T2DM patients, no matter whether they are obese or not.
